# An Uncommon Cause of Recurrent Presyncope, Dizziness, and Tachycardia: A Case Report of Diffuse, Adult-Onset Nesidioblastosis/Non-Insulinoma Pancreatogenous Hypoglycemia Syndrome (NIPHS)

**DOI:** 10.3390/biomedicines11061741

**Published:** 2023-06-17

**Authors:** Martin Philipp Dieterle, Ayman Husari, Sophie Nicole Prozmann, Hendrik Wiethoff, Albrecht Stenzinger, Manuel Röhrich, Uwe Pfeiffer, Wolfgang Rüdiger Kießling, Helena Engel, Harald Sourij, Thorsten Steinberg, Pascal Tomakidi, Stefan Kopf, Julia Szendroedi

**Affiliations:** 1 Center for Dental Medicine, Division of Oral Biotechnology, Medical Center—University of Freiburg, Faculty of Medicine, University of Freiburg, Hugstetterstr. 55, 79106 Freiburg, Germany; 2 Center for Dental Medicine, Department of Orthodontics, Medical Center—University of Freiburg, Faculty of Medicine, University of Freiburg, Hugstetterstr. 55, 79106 Freiburg, Germany; 3 Medical Center—University of Freiburg, Faculty of Medicine, University of Freiburg, Hugstetterstr. 55, 79106 Freiburg, Germany; sophie.prozmann@uniklinik-freiburg.de; 4 Institute of Pathology, Heidelberg University Hospital, 69120 Heidelberg, Germany; hendrik.wiethoff@med.uni-heidelberg.de (H.W.); albrecht.stenzinger@med.uni-heidelberg.de (A.S.); 5 Department of Nuclear Medicine, University Hospital Heidelberg, 69120 Heidelberg, Germany; 6 Pfalzklinikum for Psychiatry and Neurology AdÖR, Weinstr. 100, 76889 Klingenmünster, Germany; 7 Independent Researcher, 72250 Freudenstadt, Germany; 8 Cancer Immune Regulation Group, German Cancer Research Center, Im Neuenheimer Feld 280, 69120 Heidelberg, Germany; 9 Division of Endocrinology and Diabetology, Department of Internal Medicine, Medical University of Graz, 8010 Graz, Austria; 10 Interdisciplinary Metabolic Medicine Trials Unit, Medical University of Graz, 8036 Graz, Austria; 11 Department of Internal Medicine I and Clinical Chemistry, University of Heidelberg, 69120 Heidelberg, Germany

**Keywords:** nesidioblastosis, hyperinsulinism, hypoglycemia, hyperinsulinemic hypoglycemia, congenital hyperinsulinism, positron emission tomography, insulinoma, pancreatectomy, hyperplasia

## Abstract

Neurovegetative and autonomic symptoms are common presentations of various diseases, ranging from psychosomatic to severe organic disorders. A 23-year-old man presented with a history of recurrent presyncope, dizziness, and tachycardia. Repeated diagnostic work-up in various clinical settings could not identify any definite cause for approximately eight years. However, the incidental detection of postprandial and exercise-induced hypoglycemia was suggestive of an insulin-related disorder. A 72 h plasma glucose fasting test revealed endogenous hyperinsulinism. Upon imaging studies, no tumor mass potentially indicating insulinoma could be detected. ^68^Ga-DOTA-Exendin-4 PET/CT showed diffuse tracer enrichment throughout the whole pancreas. A subtotal pancreatectomy was performed, and the diagnosis of diffuse, adult-onset nesidioblastosis was established histopathologically. This corresponds to the clinical findings of a functional β-cell disorder, also known as non-insulinoma pancreatogenous hypoglycemia syndrome (NIPHS). After nine months, the symptoms recurred, making complete pancreatectomy necessary. Postoperative laboratory evaluation exhibited no residual endogenous C-peptide production. This case illustrates the diagnostic challenges in patients presenting with unspecific, neurovegetative and autonomic symptoms with a severe and rare underlying cause.

## 1. Introduction

The differential diagnosis of patients presenting with unspecific, neurovegetative and autonomic symptoms is complex and involves a variety of diseases, including psychosomatic, neurologic, cardiologic, and endocrine disorders [1,2]. Uncommon presentations of frequent diseases, as well as (un)common presentations of rare diseases, remain a major diagnostic challenge in clinical practice. Therefore, a thorough and complete diagnostic work-up is essential to minimize misdiagnosis and to avoid overlooking potentially life-threatening conditions [3]. The incidental detection of an additional symptom is sometimes key to finding the right diagnosis. We here report the case of a 23-year-old man, who primarily presented with unspecific autonomic symptoms, ranging from presyncope and dizziness, to recurrent and severe supraventricular tachycardia. The repeated clinical evaluation of the patient by experts from various medical specialties did not reveal any significant abnormal findings. However, incidental blood glucose measurements by the patient himself uncovered recurrent postprandial and exercise-induced hypoglycemia. This symptom finally led to the correct diagnosis of a functional β-cell disorder, also known as diffuse adult-onset nesidioblastosis or non-insulinoma pancreatogenous hypoglycemia syndrome (NIPHS) [4,5,6].

The differential diagnosis of hypoglycemia in adult patients can be challenging [7,8]. Apart from severe diseases like liver, kidney or heart failure, the intake of alcohol, insulin, or insulin secretagogues needs to be ruled out [7,9,10]. Genuine endocrine diseases, like pituitary or adrenal insufficiency, should also be considered especially if patients present with other symptoms suggestive of these diseases [11,12]. Organic hyperinsulinism, i.e., the inadequate, endogenous overproduction of insulin, is mostly caused by insulinoma. The latter is a neoplastic disease originating from the β-cells of the islets of Langerhans with an incidence of approximately 1 of 250,000 people per year. Approximately 85% of insulinomas are benign and the most common symptom is fasting hypoglycemia. Above all, due to the anabolic effects of insulin, most patients diagnosed with these disease present with a significant weight gain. The gold standard for diagnosing insulinomas is the proof of hyperinsulinemic hypoglycemia in a prolonged 72 h fasting test [13]. If clinical findings are suggestive of insulinoma but no tumor can be localized upon conventional imaging, rarer causes of recurrent spontaneous hypoglycemia in the adult patient must be considered: insulin autoimmune syndrome, also known as Hirata’s disease, as well as functional β-cell disorders [14,15]. The latter are a group of diseases also known as adult-onset nesidioblastosis or non-insulinoma pancreatogenous hypoglycemia syndrome (NIPHS) [5,16,17,18,19,20].

Historically, nesidioblastosis represents a histopathological finding, which describes the budding or neoformation of endocrine islets cells from the exocrine ducts of the pancreas [21]. In subsequent years, diffuse adult-onset nesidioblastosis has also been used to describe the clinical findings associated with functional disorders of the islets of Langerhans. To standardize diagnostic criteria, Anlauf and co-workers defined major (obligate in every case) and minor (might not be found in every patient) criteria for adult-onset nesidioblastosis in 2005. Major criteria include the macroscopic, microscopic, and immunohistochemical exclusion of insulinoma, enlarged and hyperchromatic nuclei, as well as the prominent clear cytoplasm of the β-cells in the islets of Langerhans, a regular relative and spatial distribution of the endocrine cell types in the pancreas (meaning A/α, B/β, D, and PP cells), and no significant proliferative activity of these cells (i.e., Ki-67 index < 1 %). Apart from that, minor criteria comprise islet hypertrophy, islet hyperplasia, lobulated islets, and macronucleoli within the β-cells [22,23,24]. Clinically, adult-onset nesidioblastosis presents with neuroglycopenic as well as adrenergic symptoms related to hypoglycemia. These include impaired consciousness, visual, sensorimotor and speechabnormalities, headaches, seizures, anxiety, tremors, tachycardia, sweating, and many more [25]. From a diagnostic point of view, Service et al. described the common findings associated with nesidioblastosis in 1999, and defined the term NIPHS for the clinical syndrome [26]. It comprises postprandial neuroglycopenic symptoms with documented hyperinsulinemic hypoglycemia, a negative 72 h fasting test, negative conventional imaging studies (MRI, CT), as well as a positive selective arterial calcium stimulation test with hepatic venous sampling (SACS) [26,27]. In particular, the finding of postprandial hypoglycemia in the absence of fasting hypoglycemia was long thought to be a major feature discriminative from insulinoma. However, not all cases with histologically proven nesidioblastosis exhibit the same clinical pattern. Some patients, e.g., have a positive fasting test and also suffer from fasting or exercise-induced hypoglycemia [18,28,29]. Therefore, NIPHS/nesidioblastosis rather represents a spectrum of related disorders with common and repeated, but not exclusive, clinical and histopathological findings.

The following case illustrates the diagnostic challenges associated with this rare disease and underscores the importance of careful differential diagnosis in patients presenting with unspecific symptoms. We additionally performed an extensive review of the literature concerning nesidioblastosis, islet cell hyperplasia, and related morphological characteristics in adults, and studied their relationship to hypoglycemia and other clinical presentations. This literature review is presented in a separate publication (Dieterle et al., 2023, manuscript submitted to *Biomedicines*).

## 2. The Case

The 23-year-old patient presented with a history of unspecific, neurovegetative symptoms in the Department of Endocrinology at the Heidelberg University Hospital. The symptom onset could be traced back to episodes of severe supraventricular tachycardia starting eight years prior. These episodes occurred several times a day and were accompanied by presyncope, dizziness, blurred vision, nausea, urgent urination, shortness of breath, joint pain, muscle pain, and muscle weakness. Back then, an expired myocarditis was suspected. Symptomatic treatment attempts with bisoprolol, ivabradine, or a combination of ivabradine and metoprolol were unable to mitigate the symptoms. When presenting at the emergency department of a local hospital during one acute episode, slight hypokalemia (3.37 mmol/L) was documented. Repeated diagnostic work-up, including laboratory examinations and presentations in the pulmonology, cardiology, gastroenterology, rheumatology, endocrinology, and neurology departments, was inconspicuous except for inappropriate sinus tachycardia, slight restrictive and obstructive lung disease, increased breathing, gastro-esophageal reflux disease, and a history of recurrent acute *Mycoplasma pneumonia* infections during adolescence. The latter were treated with clarithromycin and some of the above-described symptoms (especially joint and muscle pain, as well the respiratory abnormalities) were attributed to these infections.

The familial anamnesis was inconspicuous for any endocrine disease except for hypothyroidism. The consumption of alcohol or illegal drugs was denied by the patient. A review of the patient’s medical history by the pediatrician revealed an elevated leucine plasma level in the early postnatal period (4.0 mg/dL), which normalized spontaneously. At the age of 7, a (fasting?) blood glucose of 36 mg/dL had been registered, whose significance is unclear since it was not further investigated and the preanalytical process could not be reconstructed.

Of interest, the patient reported that the above-described episodes occurred more frequently after food intake, physical exercise, or a combination of both. Thus, attempts at stamina training resulted in multiple episodes of presyncope, usually after 15 min of exercise. This unusual exercise intolerance and the association with meals led to the hypothesis of a disorder of energy metabolism. Blood glucose measurements performed by the patient shortly (approximately 30 min) after meals reproducibly yielded unexpectedly low values in the range of 40–70 mg/dL. Values in the same range were reported when the patient went for a walk within the first 2–3 h after food intake. Fasting hypoglycemia was, however, not reported. The HbA1c at that time was 5.1% (32.2 mmol/mol), corresponding to an average blood glucose of 84 mg/dL.

The patient was admitted to the endocrinology department in another university hospital in southern Germany. Screening for hepatic, renal, adrenal, and pituitary insufficiency, severe inflammation, insulin-like growth factor overproduction, or insulin/insulin receptor autoantibodies was negative. A 4 h oral glucose tolerance test (OGTT) was performed and showed conspicuous results (Table 1). Two symptomatic declines in blood glucose were monitored 60 min and 210 min after oral glucose (75 g) intake. Unfortunately, some values were not determined and therefore reactive postprandial hypoglycemia was suspected.

Nutrition counseling and a subsequent low carbohydrate diet, however, did not lead to a release from the symptoms. Consequently, a 72 h fasting plasma glucose test was performed (Table 2). Persistent C-peptide levels above 0.2 nmol/L and persistent insulin levels above 21 pmol/L after 72 h of fasting, alongside corresponding low venous blood glucose values, were interpreted as pathologic according to the classical literature on hyperinuslinemic hypoglycemia [30]. The slight increase in blood glucose between 48 h and 70 h was attributed to a residual hepatic and/or renal gluconeogenesis, meaning that the amount of insulin in the blood was not sufficient to fully suppress gluconeogenesis. The concomitant increase in insulin and C-peptide levels, however, indicated a persistent and inadequately high secretion of insulin, supporting the finding of hyperinsulinemic hypoglycemia.

As factitious or iatrogenic hypoglycemia could be ruled out, endogenous hyperinsulinemic hypoglycemia was diagnosed. A treatment with diazoxide 100 mg (1-1-1) was started. Abdominal contrast-enhanced MRI did not reveal any lesion indicating insulinoma or another neuroendocrine tumor. ^68^Ga-DOTA-Exendin-4 PET/CT, which targets glucagon-like peptide 1 (GLP-1)-expressing cells, did also not reveal a focal enrichment, but a diffuse tracer enrichment throughout the whole pancreas. Therefore, the diagnosis of (occult) insulinoma seemed unlikely. Additional SACS was not performed. Since the patient had no history of gastric bypass surgery and exhibited no signs of gastroparesis or dumping-like symptoms, post-gastric bypass hypoglycemic syndrome could be ruled out. Instead, these results pointed in the direction of an adult form of a functional β-cell disorder, also called non-insulinoma pancreatogenous hypoglycemia syndrome (NIPHS).

**Figure 1:**
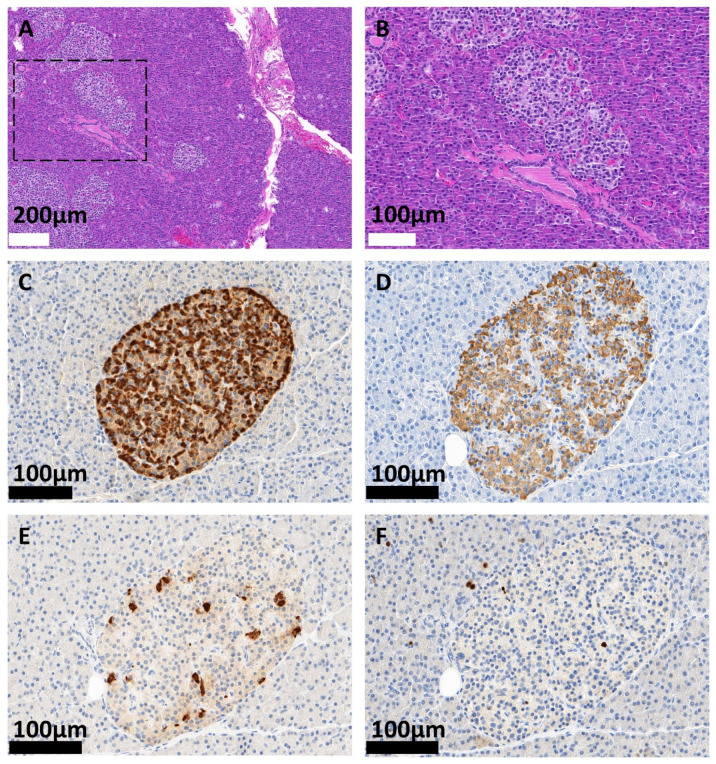
Hematoxylin/Eosin stained (HE), as well as immunohistochemically stained (IHC), histological sections of the surgical specimen. (**A**) Enlarged and variably sized and shaped islets of Langerhans are visible (lower left and dashed area), which are embedded in exocrine pancreatic tissue (HE). The dashed area is presented in (**B**). The islets contain enlarged cells, with round to oval hyperchromatic nuclei. Nucleoli are rather inconspicuous. (**C**,**D**) Physiological distribution of the neuroendocrine cell subtypes confirmed by the IHC staining of glucagon (**C**), insulin (**D**), and somatostatin (**E**). (**F**) The proliferation rate is less than 1% (Ki-67). Scale bars represent the indicated lengths.

Upon diazoxide treatment, the patient became hypotonic, tachycardic, and developed angina pectoris, resulting in a non-ST-segment elevation myocardial infarction (NSTEMI). Treatment with diazoxide was stopped and the subcutaneous application of octreotide (three times per day) was started. The therapy regimen was continued with the long-acting release form of lanreotide. However, hypoglycemic events became more frequent upon treatment with the somatostatin analogues (up to fifteen hypoglycemic events/day). The patient could not walk more than 100 m anymore without suffering from hypoglycemia, which led to his presentation at the Heidelberg University Hospital. Screening for congenital metabolic disorders presenting with hypoglycemia was negative. A physical examination of the patient revealed no abnormalities and his body mass index upon admission was 19.75 kg/m^2^. Since conservative treatment was unsuccessful, the patient underwent a subtotal, left-sided, spleen-preserving pancreatectomy. The postoperative course was complicated by pancreatic fistula, which was treated conservatively, and delayed gastric emptying due to postoperative pyloric stenosis. Histopathological examination revealed the diffuse involvement of the pancreas by variably sized and shaped pancreatic islets containing enlarged neuroendocrine cells with round to oval hyperchromatic nuclei with dense nuclear chromatin and inconspicuous nucleoli. Immunohistochemistry demonstrated a physiological distribution of insulin-, glucagon-, and somatostatin-producing cells within the islets, with a proliferation rate < 1% (Ki-67). A neuroendocrine tumor was not detected. The exocrine pancreas showed a regular histoarchitecture. In conclusion, these findings are compatible with NIPHS/nesidioblastosis according to the diagnostic criteria described above (Figure 1) [14,15].

**Figure 2:**
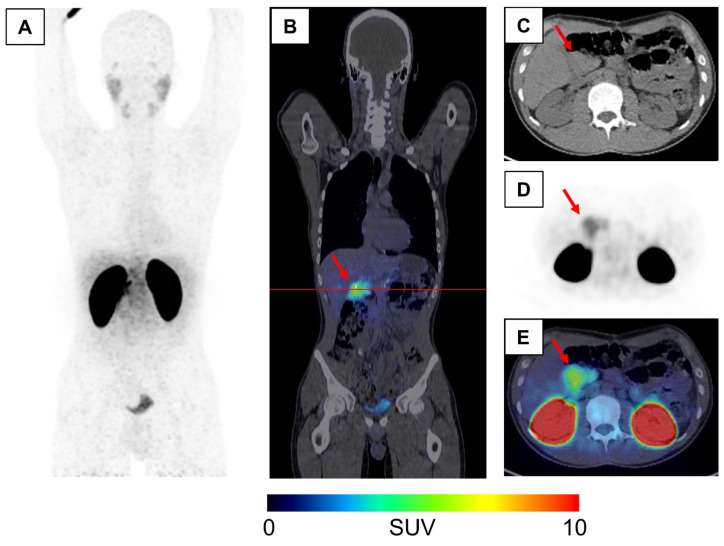
^68^Ga-Exendin PET/CT post left pancreatic resection: Intense tracer accumulation in the remaining pancreatic head led to the decision to complete the pancreatectomy via the Whipple procedure. (**A**) Maximum intensity projection (MIP) of ^68^Ga-Exendin-PET, (**B**) Coronar-fused ^68^Ga-Exendin-PET/CT image, (**C**–**E**) Axial CT (**C**), PET (**D**) and fused (**E**) ^68^Ga-Exendin-PET/CT images at the height of the pancreatic head (indicated by red line). The red arrow indicates the remaining PET-positive pancreatic head. PET/CT images were windowed using the following parameters: CT: center 50 HU, width 400 HU; PET: 0 (black)—10 (intense red) standardized uptake value (SUV).

After nine months, symptomatic hypoglycemia reappeared, which also became apparent since the patient used a continuous glucose monitoring (CGM) system. Extra-pancreatic insulin production was ruled out with ^68^Ga-DOTA-Exendin-4 PET/CT, which only revealed diffuse tracer enrichment in the residual pancreatic head with a maximum standardized uptake value (SUV_max_) of 10.26 (Figure 2). A complete pancreatectomy (classical Whipple procedure) was performed, and histopathological examination again confirmed a pattern typical of diffuse, adult nesidioblastosis. On the 11th postoperative day, the patient developed a severe hemorrhagic shock due to the Forrest Ib bleeding of an ulcer in the gastroenterostomy. Diabetic control was further complicated by early dumping syndrome and severe nausea.

Two years postoperatively, no endogenous insulin production could be detected, as revealed by C-peptide levels below 0.1 ng/mL, which confirms the complete removal of all endocrine pancreatic tissue. Due to cachexia, partial parenteral nutrition is still needed (950 kcal/day). The type 3c diabetes is well controlled with a combination of regular (insulin correction factor: 50 mg/dL, insulin-to-carb ratio: 1:10) and NPH insulin (three times daily; 4 IU [6 a.m.], 3–4 IU [2 p.m.], 3–4 IU [10 p.m.]). According to the American Diabetes Association (ADA), type 3c diabetes, also known as pancreoprivic diabetes, is defined as a special form of diabetes caused by a global dysfunction of the entire pancreas, as seen in acute or chronic pancreatitis, cystic fibrosis, pancreatic neoplasia, pancreatic trauma, or the partial/total surgical removal of the pancreas (as in this case) [31]. Due to the reduction in or loss of functional endocrine pancreatic tissue, i.e., mainly insulin, glucagon, somatostatin, and pancreatic polypeptide-secreting cells, it is pathophysiologically and clinically different from type 1 diabetes. Diabetes type 3c is mainly characterized by an extraordinary insulin sensitivity, which can be explained in terms of the missing counterregulatory glucagon [32,33,34,35,36]. Patients suffering from this type of diabetes have a high susceptibility to hypoglycemia since the autonomic symptoms of hypoglycemia can be missing. In the case of total pancreatectomy, which leads to a total loss of glycemic control, 1 IU of insulin can lead to a decrease in blood glucose of 50–70 mg/dL (personal experience; insulin correction factor [see above]; this is compared to 30–40 mg/dL in type 1 diabetes) depending on the time of day, which makes the management of type 3c diabetes challenging. A current review article by Zhao and colleagues provides an in-depth discussion of this topic [35]. Currently, the HbA1c of our patient is 6.6% [59.6 mmol/mol], corresponding to an average blood glucose of 143 mg/dL with a time in range (70–180 mg/dL) of approximately 70%, as documented by the CGM device. Since hospital discharge, no severe hypoglycemic events have been reported.

## 3. Discussion

The diagnostic work-up of patients presenting with unspecific, autonomic symptoms is very complex, and distinguishing harmless functional disorders from severe organic dysfunctions can be challenging. Rare diseases should be considered in patients if they present with progressive and/or additional symptoms. The presented case shows that the careful interpretation of symptoms and their correlation with daily activities can finally lead to the correct diagnosis. Of interest, the slight hypokalemia detected during a symptomatic episode was the only initial laboratory finding that could have indicated hyperinsulinism, before actual hypoglycemia was recognized.

**Figure 3:**
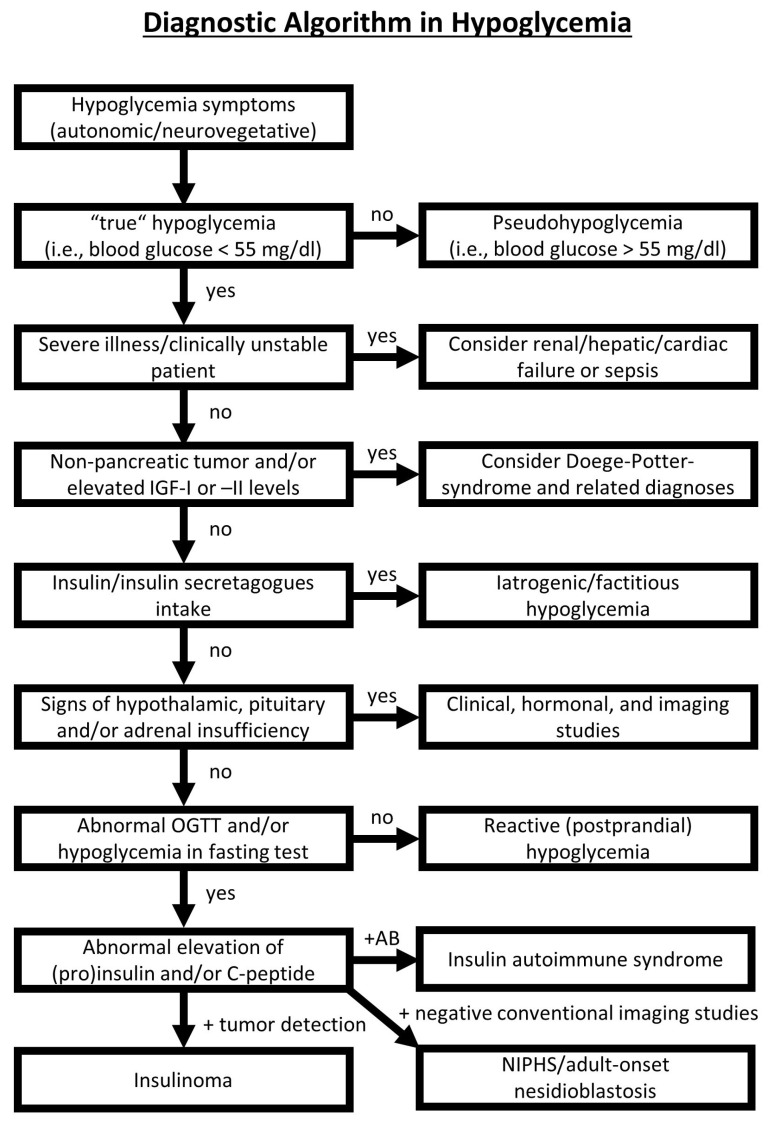
Diagnostic approach to the adult patient presenting with spontaneous hypoglycemia. First, hypoglycemia must be proven via blood glucose measurement (preferably by intravenous blood sampling). “True” hypoglycemia is defined by the Whipple triad: blood glucose < 55 mg/dL, symptoms of hypoglycemia, and a relief of symptoms upon glucose intake. It these criteria are not fulfilled, pseudohypoglycemia is suspected (also known as idiopathic postprandial syndrome or adrenergic postprandial syndrome). In the next step, severe illness must be ruled out. As hypoglycemia can be caused by renal, hepatic, or cardiac failure as well as sepsis, a critically ill patient with diagnosed hypoglycemia needs rapid diagnostic work-up. In other cases, non-pancreatic tumors, such as fibrous tumors of the thorax (e.g., Doege–Potter Syndrome), need to me considered since they can lead to the paraneoplastic secretion of insulin-like growth factors, which provoke hypoglycemia. Factitious hypoglycemia is induced by the inadequately high application of insulin or insulin secretagogues such as sulfonyl urea. A lack of growth hormone release hormone (GHRH), growth hormone (GH), corticotropin-releasing hormone (CRH), adrenocorticotropic hormone (ACTH), or cortisol can also lead to hypoglycemia. In the absence of hormonal gland insufficiencies, oral glucose tolerance testing (OGTT) can further help to differentiate reactive (postprandial) hypoglycemia (low plasma glucose with adequately low insulin and C-peptide levels) from (endogenous) hyperinsulinemic hypoglycemia. The latter is either caused by insulinoma (clinically mostly associated with fasting hypoglycemia and a pancreatic or ectopic insulin-producing neoplasia upon imaging studies), insulin autoimmune syndrome (also called Hirata´s disease, defined by insulin or insulin receptor autoantibodies upon laboratory investigation), or NIPHS/adult-onset nesidioblastosis (clinically often defined by postprandial or exercise-induced hypoglycemia and no detectable tumor mass upon conventional imaging studies). More details concerning the differential diagnosis of hypoglycemia can be found in our corresponding review article (Dieterle et al. 2023, manuscript submitted to *Biomedicines*) and the following references [7,42,43]. IGF-I or -II = insulin-like growth factor I or II; OGTT = oral glucose tolerance test; AB = insulin and/or insulin receptor autoantibodies; NIPHS = non-insulinoma pancreatogenous hypoglycemia syndrome.

Hypoglycemia in the non-diabetic adult patient needs a thorough clinical and laboratory work-up [4]. We propose a systematic approach to the patient, as presented in Figure 3 (details are discussed in the figure legend). If hyperinsulinemic hypoglycemia is suspected, a prolonged (4–6 h) oral glucose tolerance test (OGTT) and a 72 h fasting test should be performed. Above all, other rare conditions such as Hirata’s disease (insulin autoimmune syndrome) or Doege–Potter syndrome should be excluded via laboratory methods [14,37]. The conventional sectional imaging of the abdomen helps to differentiate insulinoma from functional β-cell disorders. While SACS was the gold standard in the past to detect an insulin gradient in the vessels supplying the pancreas, this invasive method might be almost completely replaced by PET imaging in the future, e.g., with ^68^Ga DOTA-Exendin-4 PET/CT [38,39,40,41]. The latter is especially helpful in distinguishing occult or ectopic insulinoma from NIPHS and to separate focal and diffuse nesidioblastosis [29].

The most frequent underlying cause of hyperinsulinemic hypoglycemia in the adult patient is (benign) insulinoma [44]. Idiopathic/sporadic adult-onset nesidioblastosis with hyperinsulinemic hypoglycemia is an extremely rare disease that occurs much less frequently. It is estimated that about 1 in 10,000,000 people are diagnosed with this disease annually [45,46,47,48].

While the pathophysiology of persistent hyperinsulinemic hypoglycemia in childhood and infancy, which is morphologically similar to NIPHS, is well understood from a genetic and functional point of view, mechanistic data on adult-onset NIPHS is scarce [49]. Some experimental evidence suggests that a higher basal insulin secretion, as well as changes in the resting membrane potential of β-cells, might contribute to the biochemical and clinical findings of the disease [50,51,52]. Of note, the histologic and clinical changes in exhibited in patients with NIPHS differ considerably. There is, however, currently no established hypothesis to explain the morphological differences between patients or to correlate the morphological findings with clinical presentations [22,24]. While many patients with NIPHS report postprandial hypoglycemia, others present with exercise-induced hypoglycemia or a combination of both [28]. In some cases, fasting hypoglycemia was also reported in NIPHS. The latter point is especially important since it was commonly assumed that fasting hypoglycemia is a hallmark of insulinoma and that a positive fasting test rules out the diagnosis of NIPHS. As can be seen from our case and other reports in the literature, a positive fasting test neither confirms insulinoma nor excludes NIPHS [53,54]. Above all, exercise-induced or postprandial hyperinsulinemic hypoglycemia is not a defining feature of NIPHS, since some insulinoma patients also suffer from these symptoms [55,56,57]. Taken together, the clinical and laboratory findings in our patient were neither exclusively suggestive of insulinoma nor of a functional β-cell disorder. The presence of fasting hypoglycemia during the 72 h fasting test (although recognized very late during the test) and concomitant postprandial and exercise-induced hypoglycemia made it necessary to definitely exclude an insulin-producing neoplasia. Since PET/CT imaging rendered occult insulinoma very unlikely, the clinical diagnosis of NIPHS was justified even without SACS testing. However, the histopathological confirmation of the diagnosis, as beautifully illustrated by Figure 1, still is the gold standard in NIPHS/diffuse adult-onset nesidioblastosis. The strict criteria of Anlauf and colleagues should be applied (see above). Once the diagnosis of nesidioblastosis/NIPHS is suspected, treatment options need to be discussed with the patient. While a low carbohydrate diet or α-glucosidase inhibitors are not effective in most patients, the potassium channel activator diazoxide has been shown to alleviate symptoms in some cases [58,59]. However, severe adverse effects have been reported in this context, which is also illustrated by the presented case. Some authors also proposed the use of Calcium channel antagonists such as Verapamil to reduce insulin secretion from the pancreas [60]. Hypotension is a limiting factor in the use of these drugs. The stimulation of somatostatin receptors by octreotide, lanreotide, or pasireotide has also repeatedly been described in the literature in the context of NIPHS. Since these substances also inhibit the secretion of glucagon, symptoms of hypoglycemia may occur even more frequently in a subset of patients [52,61,62]. Schwetz et al. discussed this phenomenon in the context of the different binding affinities of the respective somatostatin analogues to the different somatostatin receptor subtypes [62]. Insulin secretion is reduced by somatostatin receptor type 2 and 5 signaling, while glucagon secretion is mainly inhibited by receptor type 2 signaling. Since octreotide and lanreotide mainly bind somatostatin receptor subtype 2, the substances both inhibit insulin and glucagon secretion, which might precipitate hypoglycemia. Contrary to that, pasireotide has the highest affinity for somatostatin receptor subtype 5 and was effective in at least one NIPHS patient with hypoglycemia resistant to octreotide treatment [62].

In most cases, surgical intervention is the only definitive approach to reduce the frequency and intensity of hypoglycemic events and simultaneously allows for a definitive histological diagnosis. There is, however, a controverse discussion regarding the extent of pancreatic resection in cases of NIPHS. While some patients are relieved from all symptoms upon left-sided partial/subtotal pancreatectomy, others need to undergo total pancreatectomy for symptom control [63,64,65]. Of note, surgical procedures on the pancreas, especially total pancreatectomy, are associated with a considerable risk of morbidity and mortality [33,66,67,68]. The management of type 3c diabetes, with all its difficulties and potential complications, as described above, is a long-term challenge for caregivers. In the pre-CGM era, the total loss of glycemic control was associated with severe complications in patients undergoing total pancreatectomy [35,66,69].

As the presented case shows, the rapid onset of new hypoglycemic events after partial pancreatectomy is indicative of too much residual insulin-secreting pancreatic tissue. Of note, this could be correlated with significant tracer uptake upon imaging using ^68^Ga-DOTA-Exendin-4 PET/CT. This finding is especially interesting in the context of new therapy options for NIPHS/adult-onset nesidioblastosis in the future. In light of the potentially life-threatening complications of pancreatic surgery, minimally invasive procedures targeting the pancreatic β-cells are a promising approach. Boss and colleagues presented cell culture and mouse in vivo data on selective destruction of GLP-1-expressing cells via targeted photodynamic therapy. Although initially aimed at treating congenital hyperinsulinism of infancy and childhood, this elegant approach could also be applied to NIPHS in order to overcome surgical interventions. A major challenge remains the adequate dosage, which guarantees relief from hypoglycemia while simultaneously preserving the other endocrine and exocrine functions of the pancreas [70].

## 4. Conclusions

Diffuse adult-onset nesidioblastosis is a rare disorder presenting with hyperinsulinemic hypoglycemia and various clinical symptoms, which can easily be misinterpreted as, e.g., anxiety disorder. Thorough clinical work-up is essential to avoid overlooking this potentially life-threatening disease. Medical treatment includes a low-carbohydrate diet, alpha-glucosidase inhibitors, diazoxide, calcium channel inhibitors, and somatostatin analogues, but is only effective in a small subset of patients. The surgical resection of parts or even the entire pancreas is often the only chance to effectively reduce or abolish hypoglycemic events. This is, however, accompanied by a considerable risk of morbidity and mortality, as illustrated by the presented case. Novel imaging modalities like ^68^Ga-DOTA-Exendin-4 PET/CT are helpful in distinguishing occult insulinomas from nesidioblastosis/NIPHS and help to avoid surgical intervention that is unnecessary or too expansive. The presented case reflects the many facets of and difficulties in diagnosing and treating this complex and rare disease. A concise review and discussion of all aspects of adult-onset nesidioblastosis/NIPHS is given in our review article (Dieterle et al. 2023, submitted to *Biomedicines*), and the interested reader is directed to this publication for further insights into this intriguing disease.

## Figures and Tables

**Table 1:** Results of the 4 h oral glucose tolerance test.

	0 min	30 min	60 min	90 min	120 min	180 min	210 min	240 min
Glucose mg/dL	87	132	78	122	103	82	50	73
Insulin pmol/L	51	Not determined	508	328	792	239	Not determined	32
C-peptide nmol/L	0.52	Not determined	3.08	2.57	3.84	2.38	Not determined	0.68

**Table 2:** Results of the 72-h fasting plasma glucose test.

	48 h Fasting	70 h Fasting	72 h Fasting
Glucose mg/dL	56	57	48
Insulin pmol/L	34	65	31
Insulin mIU/L	4.9	9.7	4.5
C-peptide nmol/L	0.26	0.47	0.32

## Data Availability

The data presented in this study are available on request from the corresponding author. The data are not publicly available due to privacy issues (single case report).
